# Transcriptional changes in specific subsets of *Drosophila* neurons following inhibition of the serotonin transporter

**DOI:** 10.21203/rs.3.rs-2626506/v1

**Published:** 2023-03-17

**Authors:** Shivan L. Bonanno, David E. Krantz

**Affiliations:** 1 Department of Psychiatry and Biobehavioral Sciences, David Geffen School of Medicine, University of California, Los Angeles, CA 90095, USA

## Abstract

The transcriptional effects of SSRIs and other serotonergic drugs remain unclear, in part due to the heterogeneity of postsynaptic cells, which may respond differently to changes in serotonergic signaling. Relatively simple model systems such as *Drosophila* afford more tractable microcircuits in which to investigate these changes in specific cell types. Here, we focus on the mushroom body, an insect brain structure heavily innervated by serotonin and comprised of multiple different but related subtypes of Kenyon cells. We use fluorescence activated cell sorting of Kenyon cells, followed by either or bulk or single cell RNA sequencing to explore the transcriptomic response of these cells to SERT inhibition. We compared the effects of two different *Drosophila* Serotonin Transporter (*dSERT*) mutant alleles as well as feeding the SSRI citalapram to adult flies. We find that the genetic architecture associated with one of the mutants contributed to significant artefactual changes in expression. Comparison of differential expression caused by loss of SERT during development versus aged, adult flies, suggests that changes in serotonergic signaling may have relatively stronger effects during development, consistent with behavioral studies in mice. Overall, our experiments revealed limited transcriptomic changes in Kenyon cells, but suggest that different subtypes may respond differently to SERT loss-of-function. Further work exploring the effects of SERT loss-of-function in other *Drosophila* circuits may be used help to elucidate how SSRIs differentially affect a variety of different neuronal subtypes both during development and in adults.

## Introduction

Though serotonergic neurons comprise only ~1/200,000 neurons in humans, they project to and influence nearly every region of the mammalian brain [1,2], and represent a commonly targeted neurotransmitter system in the treatment of depression [3–6]. The predominant method by which serotonin is cleared from the extracellular space is through reuptake into the presynaptic cell by the plasma membrane serotonin transporter (SERT) [7–10]. SERT is the target of Selective Serotonin Reuptake Inhibitors (SSRIs), which inhibit its activity and thus prolong the availability of extracellular serotonin to bind and activate serotonin receptors (5-HTRs). Widespread prescription of these drugs has motivated many studies of their long-term effects utilizing peripheral samples [11–13] or highly heterogeneous brain tissue [14,15]. However, deeper understanding of serotonergic circuits and their responses to therapeutic interventions remains elusive due in part to the heterogeneity of serotonergic neurons themselves and the cells that they innervate. Such cellular diversity has been highlighted recently in mammals [1,16,17], and a few studies have analyzed gene expression in specific populations of cells postsynaptic to serotonergic neurons [18,19]. Several reports have investigated changes in ribosome-loaded RNA in a particular cell-type after environmental/behavioral perturbations and/or SSRI administration [20,21]. Another recent study has generated multi-omic datasets on fluoxetine vs. sham-treated mice across multiple brain regions, including two datasets utilizing scRNA-seq to analyze specific hippocampal cell types [14]. The complexity of these findings suggests that further, detailed analysis of the response that occurs in different subtypes of neurons will be necessary to fully understand the molecular effects of SERT inhibition.

Similar to the mammalian CNS, the *Drosophila* brain is innervated by relatively few (~90) broadly projecting serotonergic neurons [22–24]. Due to its relative simplicity, it is much easier to identify structures and circuits in the *Drosophila* brain that are innervated by one or a few, particular serotonergic neurons. This, coupled with the genetic tools available in flies, affords a technically tractable platform for the molecular interrogation of serotonergic circuits and in particular, specific subsets of post-synaptic neurons that receive serotonergic inputs.

The mushroom bodies (MBs) are structures in the central brain of *Drosophila* and other insects required for learning as well as other behaviors [25]. They are densely innervated by a small number of serotonergic cells [26–31] and are comprised of three major cell subtypes of Kenyon cells (KCs) including α/β, α’/β’, and γ KCs, which can be further subdivided based on morphology, birth order, and gene expression [32,33]. The three major KC subtypes are known to differ in 5-HTR expression profiles [33,34], with 5-HT1A enriched in KC_α/β_ and 5-HT1B in KC_γ_ proposed to regulate different behavioral outputs [35–37].

We have employed bulk RNA-seq as well as single cell RNA-seq following the isolation of KCs, and identify a small number of genes are differentially expressed in the MBs following inhibition of SERT activity. Our results also highlight several technical considerations relevant to the further transcriptional studies of serotonergic circuits.

## Methods

### Fly husbandry and genetic lines

Flies were maintained on a standard cornmeal and molasses-based agar media with a 12:12 hour light/dark cycle at room temperature (22–25°C).

For experiments involving drug-induced SERT blockade (Fig. 5), female flies were sorted on the day of eclosion and maintained on 1% agar + 5% sucrose + 1% blue food dye, with or without the addition of 3mM citalopram (Sigma, St. Louis, MO, USA, PHR1640), for 4-6 days before dissection.

### Fly lines/alleles used

The following fly lines were used in this study are as follows, with stock numbers for lines obtained from the Bloomington *Drosophila* Stock Center (BDSC, Bloomington. Indiana, USA) listed in parentheses: *w*^*1118*^ (BDSC:5909), *Mef2-gal4* (BDSC:50742), *UAS-nls.GFP* (BDSC:4776), *dSERT*^*4*^ (gift from H. Schölz), *dSERT*^*16*^ (gift from H. Schölz), *dSERT*^*TMKO*^ (created in this work), DGRP-21 (BDSC:28122), DGRP-129 (BDSC:28141), DGRP-235 (BDSC:28275), DGRP-304 (BDSC:25177), DGRP-320 (BDSC:29654), DGRP-324 (BDSC:25182), DGRP-354 (BDSC:55020), DGRP-382 (BDSC:28189), DGRP-383 (BDSC:28190), DGRP-395 (BDSC:55022), DGRP-406 (BDSC:29657), DGRP-437 (BDSC:25194), DGRP-461 (BDSC:28200), DGRP-819 (BDSC:28242).

### FACS and RNA-seq library preparation

**Figure 1.**
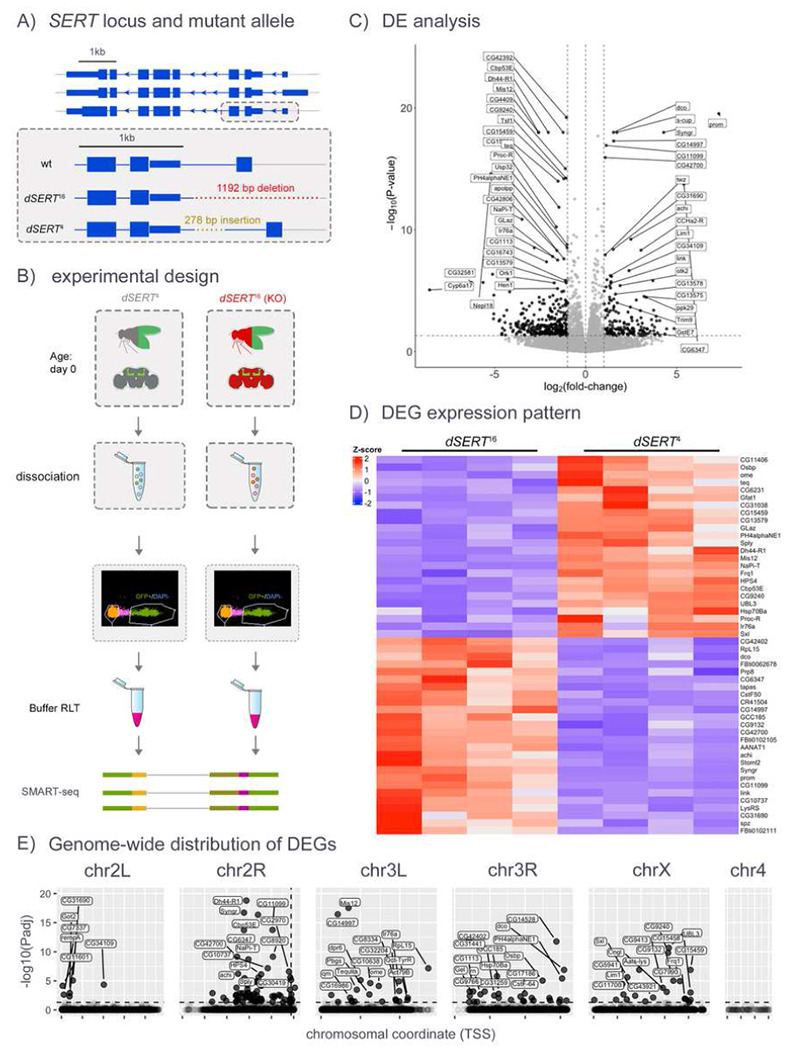
bulk RNA-seq of KCs, in immediately-eclosed (day 0) flies. A) The *Drosophila* dSERT locus encodes three transcripts (top panel). The *dSERT^16^* mutant bears a 1.1 kb deletion at the 5’ end that includes a non-coding exon and upstream regulatory DNA. The *dSERT^4^* genetic background-matched control contains a 278 bp deletion but does not significantly alter protein expression or behavior compared to WT [62], B) Sample preparation for bulk sequencing. Flies contained the *Mef2(P247*)-*gal4* driver and *UAS-nls.GFP* marker for expression in KCs, and were homozygous for either *dSERT^16^* (mutant) or *dSERT^4^* (control) on the second chromosome. Flies were dissected and pooled by genotype, then dissociated and FACS-sorted in parallel to select for GFP-labeled KCs, followed by isolation of RNA for bulk RNA-seq (SMART-seq). C) Volcano plot showing differential expression between *dSERT^16^* and *dSERT^4^* groups. DE genes include those encoding the transcription factors Lim1 and Achi, the channels *Ork1* and *Ppk29*, the GPCRs *Dh44-R1*, *Proc-R*, *CCHa2-R*, and *Ir76a*, the calcium binding protein *Cbp53E*, and genes implicated in neuronal development (*Trim9*, *Mis12*). D) The top 50 DE genes are shown as a z-score heatmap. E) DEGs plotted by chromosomal Drosophila serotonin coordinates of genomic locus, with inverse log_10_(p_adj_) on they-axis. The horizontal dashed line represents p_adj_ ≤ 0.05 cutoff. Most DE genes localize to the same chromosomal arm (chr2R) as dSERT (vertical dashed line).

**Figure 2.**
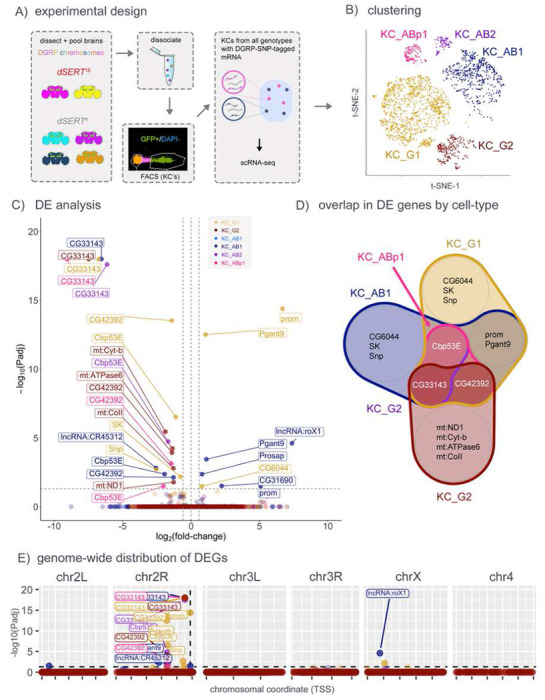
scRNA-seq of KCs from *dSERT*^16^ and *dSERT*^4^ flies, in immediately-eclosed (day 0) flies. A) Flies used for scRNA-seq contained one of six unique 3^rd^ chromosomes derived from different DGRP wild-type lines, as well as the markers and *dSERT* alleles used for bulk seq. Two and four different DGRP lines per group (*dSERT*^16^ or *dSERT*^4^, respectively) were created and served as biological replicates. Brains from all lines were dissected, pooled, and dissociated together, then FACS-sorted to select KCs used for scRNA-seq. B) t-SNE dimensional reduction showing distribution of cells in this dataset among transcriptionally-defined clusters (see methods) representing KC_g_ cells (KC_G1, KC_G2), KC_a/b_ (KC_AB1, KC_AB2), and KC_a’/b’_ (KC_ABp1). C) Volcano plot from “pseudobulk” analysis (by cluster) of DEGs between *dSERT*^16^ and *dSERT*^4^. Observations are color-coded (as in B) by the KC-type in which they were identified. D) Venn Diagram showing overlap of DEGs identified in the major cell clusters. *Cbp53E*, *CG42392*, and *CG33143* were identified as DE in multiple cell types. E) DEGs plotted by chromosomal locus as in Figure 1E. A skewed localization of DEGs to chr2R is notable.

**Figure 3.**
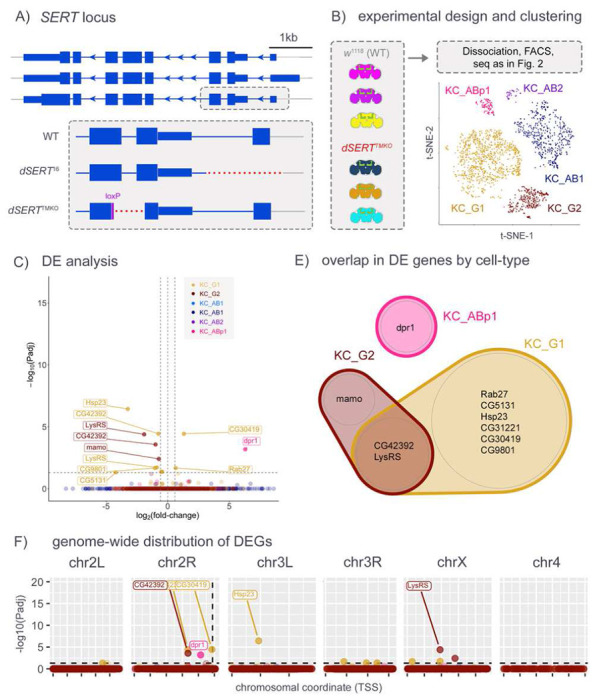
*dSERT*^TMKO^ scRNA-seq, in immediately-eclosed (day 0) flies. A) Cartoon depicts the independently-derived *dSERT*^TMKO^ deletion compared to *dSERT*^16^. B) Flies used for this scRNA-seq experiment were homozygous for *dSERT^TMKO^* or a WT *dSERT* allele derived from *w^1118^* and expressed with the same transgenes for isolation of KC cells as in Fig 1 and 2. Each fly was marked by a different DGRP 3^rd^ chromosome variant, and t-SNE plot shows the color-coded distribution of cells by KC cell-type as in Fig. 2. C) Volcano plot as in Fig. 2C from “pseudobulk” analysis (by cluster) of DEGs between mutant and control. Observations are color Drosophila serotonin coded (as in B) by the KC-type in which they were identified. D) Venn Diagram showing overlap of DEGs identified in the major cell clusters. CG42392 and LysRS were identified as DE in both KC_G1 and KC_G2. E) DEGs plotted by chromosomal position as in Fig. 2F. In contrast to Fig. 2, observations are not concentrated on chr2R.

**Figure 4.**
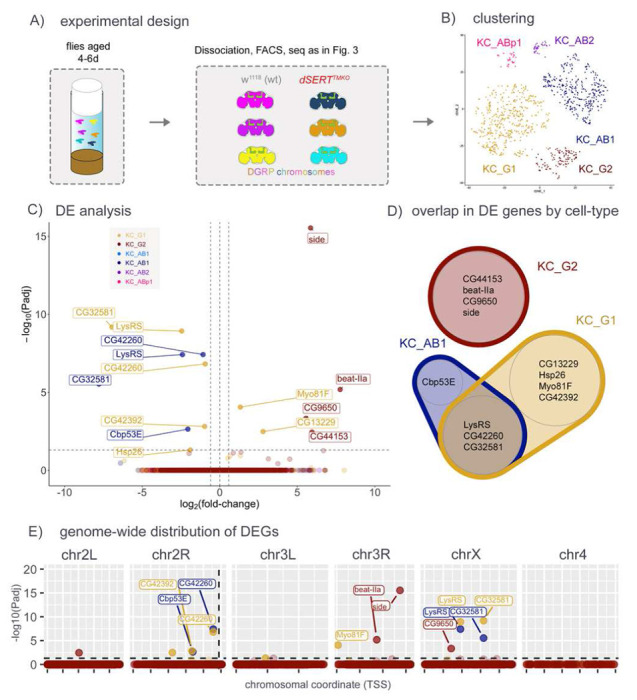
scRNA-seq for d*SERT*^TMKO^ vs controls in aged (day 4-6) flies. A) Flies harboring the dSERTTMKO or WT dSERT alleles (in the control line w1118 ) were aged for 4-6 days then processed for scRNA-seq as in Figure 3. B) t-SNE plot showing identified cell clusters color-coded by KC-type. C) Volcano plot as in Figs. 2 and 3C from “pseudobulk” analysis (by cluster) of DEGs between mutant and control. Cell-type specific DEGs include beat-lla and side in KC_G2, Myo81 F in KC_G1, Cbp53E in KC_AB1 and LysRS in KC_G1 and KC_AB1, which was also DE at day 0. D) Venn Diagram showing overlap of DEGs identified in the major cell clusters. LysRS, CG42260 and CG32581 were identified as DE in both KC_AB1 and KC_G1. E) DEGs plotted by chromosomal position as in Figs. 2 and 3F. Similar to Fig. 3 and in contrast to Fig. 2, observations are not concentrated on chr2R.

**Figure 5.**
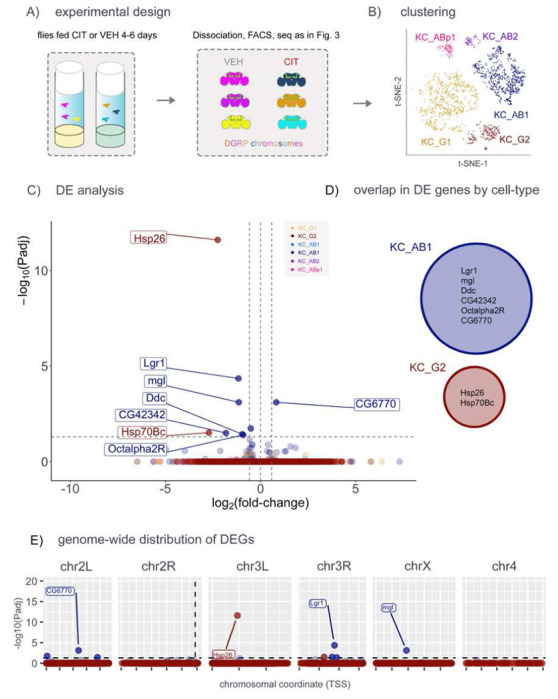
scRNA-seq in aged flies treated with an SSRI. A) Flies with WT dSERT alleles were treated with citalopram (CIT) to block SERT protein activity or vehicle alone (VEH). Each fly contained one copy of 2nd and 3rd chromosomes derived from a unique DGRP line and transgenes for marking KCs as in previous figs. B) t-SNE plot indicating the distribution of cells by cell-type. C) Volcano plot from “pseudobulk” analysis (by cluster) of DEGs between mutant and control. Cell-type specific DEGs include Lgr1 and Ddc in KC_AB1 and Hsp26 and Hsp70Bc in KC_G2, none of which were identified in previous experiments. D) Venn Diagram showing that there is no overlap of DEGs identified in the major cell clusters. E) DEGs plotted by chromosomal position as in previous figs. Similar to Figs. 3 and 4, observations are not concentrated on chr2R.

Fly lines were constructed as described, bearing *Mef2(P247)-gal4* driving *UAS-nls.GFP* to label Kenyon cell (KC) nuclei. Brains were dissected on the day of eclosion (day 0, Fig. 1–3), or day 4-6 (Fig. 4, 5) and the optic lobes removed. Central brains were pooled and dissociated according to previously published methods [38]. The dissociated brain cells were separated by fluorescence-activated cell sorting (FACS) into GFP-positive and GFP-negative isolates using a BD FACS Aria II high-speed cell sorter at the UCLA Jonsson Comprehensive Cancer Center (JCCC) and Center for AIDS Research Flow Cytometry Core Facility [26–31].

### Bulk RNA-seq

For each bulk RNA-seq replicate, 18–40 brains were dissected per genotype. Cells were collected directly off FACS (5,900–10,400 GFP^+^ cells per replicate) and lysed immediately in Buffer RLT (Qiagen #79216, Maryland, USA). RNA was purified using a commercial column (RNeasy kit, Qiagen #74034). RNA was stored at −80°C until 5 replicates were collected. Libraries for all samples were prepared simultaneously according to the SMART-seq v2 Ultra Low-input RNA sequencing kit with Nextera XT (Takara Bio, Maryland, USA, v4 #634893), using a protocol adapted from [39–41] and available upon request. Libraries were sequenced with spike-in Phi-X at the UCLA BSRC High Throughput Sequencing Core (https://stemcell.ucla.edu/high-throughput-sequencing) on an Illumina NovaSeq SP 2x50bp. After demultiplexing, 24–88 million reads per sample were retained. Quality control was performed using base metrics and nucleotide composition of raw reads. Alignment to the *Drosophila melanogaster* genome (BDGP6) was performed using the STAR spliced read aligner [42] with default parameters. Only uniquely mapped reads were used for subsequent analyses. PCA analysis showed that one pair of samples had modestly increased technical variability, and was removed from subsequent analyses. Differential expression was calculated between mutant and WT samples using DESeq2 [43].

### scRNA-seq

For each single cell RNA-seq experiment, 7–12 brains were dissected per genotype, and the genotypes pooled for subsequent processing. GFP^+^ cells representing all of the pooled samples were isolated via FACS (6500-10,000 per experiment), collected in Schneider’s media containing BSA, and transported immediately to the UCLA Technology Center for Genomics and Bioinformatics (TCGB) Core Facility (https://www.uclahealth.org/pathology/tcgb) for sample processing using the *10x Genomics* 3’ GEX v3 platform. For experiments in Fig. 2 (*dSERT*^*16*^, day 0) and Fig. 3 (*dSERT*^*TMKO*^, day 0), cells from each experiment were loaded on an individual chip from *10x Genomics*. Similarly, the cells collected experiments in Fig. 4 (*dSERT*^*TMKO*^, day 46) or Fig. 5 (CIT, day 4-6) were combined into a single sample and loaded onto a single *10x* chip thus reducing variability caused by differences in sample preparation seen in most other RNA-seq methods. For all *10x* chips, the maximum sample volume was loaded, targeting an upper limit of ~10,000 cells. cDNA and libraries were prepared and checked for size distribution by ScreenTape analysis (Agilent Technologies, Carpinteria, CA, USA). Libraries were sequenced on an *Illumina* NovaSeq SP 2x50bp. Raw sequencing reads were processed using Cell Ranger (7.0.0) with default parameters. The reference genome and gene annotations were obtained from FlyBase (6.29). Processed single-cell transcriptomes were demultiplexed based on parental genotypes using demuxlet (version 2, https://github.com/statgen/popscle) [44]. In total, genotypes of 14 DGRP strains were used for demultiplexing: DGRP-21, DGRP-129, DGRP-235, DGRP-304, DGRP-320, DGRP-324, DGRP-354, DGRP-382, DGRP-384, DGRP-395, DGRP-406, DGRP-437, DGRP-461, DGRP-819 (http://dgrp2.gnets.ncsu.edu) [45]. The genomic coordinates of variants were transformed from the dm3 to the dm6 version of the Drosophila reference genome using Crossmap [46]. The following criteria were used to filter variants used for the analysis: (1) only variants residing on chromosome 3 (see Experimental Design); (2) only biallelic single-nucleotide polymorphisms (SNPs) that were called in all analyzed DGRP strains with a maximum non-reference allele count of 2 (i.e. SNPs detected in only one of the strains); (3) the non-DGRP chromosome 3 was analyzed for SNPs that could be shared with DGRP strains, and those variants were removed from the analysis. BAM files from Cell Ranger were used to generate read pileups and to estimate allelic frequencies in our datasets. Alleles detected with high-frequency (i.e. half of the total reads deriving from the 3^rd^ chromosome) are expected to originate from the common non-DGRP chromosome. Only SNPs with minimum coverage of 5 reads and minor-allele frequencies less than 0.2 were kept for the analysis. The processing of the VCF file was performed using VCFtools [47], and SAMtools [48].

The final set included 93084 SNPs, which were transformed into heterozygous variants for the demultiplexing of F1 samples (i.e. alleles were modified from 1/1 to 1/0). The same VCF file was used for demultiplexing of all experiments. The genotypes that were not used in a particular experiment/sample were used as negative controls. Raw sequencing reads and the VCF file for demultiplexing will be available at the NCBI repository (upload to GEO in progress).

Single-cell data analysis was performed using Seurat (v4.1.1) [49,50]. Single-cell transcriptomes were filtered using the following criteria: (1) transcript count ≥ 1000; (2) maximum percentage of mitochondrial transcripts ≤ 20%; (3) we also removed cells that were classified by demuxlet as “doublets/ambiguous”, and cells that were assigned to the genotypes that were not used in the given experiment.

Filtered datasets from all three experiments were analyzed together. First, we integrated all datasets using Seurat V3 workflow with default parameters [49]. The integrated dataset was used for unsupervised clustering using the standard Seurat workflow (principal components: 1:10, resolution: 0.3). This analysis revealed 13 clusters, of which 6 expressed markers of Kenyon cells (Supp. Fig. 1A-B). We then removed non-KC clusters and re-ran integration and clustering steps (principal components: 1:10, resolution: 0.1), which yielded 8 transcriptionally distinct populations of KCs. These clusters were annotated based on known marker genes of KC subtypes (Supp. Fig. 1B-C). Three small clusters were present only in one of three experiments and were excluded from further analysis (KC_G3, KC_G4, and KC_AB3).

Differential gene expression analysis was performed for each KC cluster and each experiment separately using the “pseudobulk” approach [51]. Read counts from single-cell transcriptomes were aggregated at the level of biological replicates (i.e. DGRP strains, see Experimental Design for details). Differential analysis was performed between control and mutant/drug samples using DESeq2 [43]. Differentially expressed genes were identified at adjusted p-value (p_adj_) ≥ 0.05 and fold-change ≥ 1.5.

Data in all figures was processed and plotted using the following R packages: ggplot2 [52], tidyverse [53], ggrepel [54], patchwork [55], nVennR [56], Libra [57], DESeq2 [43], edgeR [58,59], Limma [60], and Seurat [49,50,61].

## Results

To achieve a complete loss of dSERT activity we focused our initial experiments on *dSERT* mutants rather than drug induced blockade. We used previously described flies homozygous for a *P*-element-excision-derived mutant allele (*dSERT^16^*) or a genetically-matched control (*dSERT*^*4*^) with wild-type (WT) dSERT expression [62] (Fig. 1A) and *Mef2(P247)-gal4* [63] driving nuclear-localized GFP to label Kenyon cells. This driver captures most of the KCs across all 3 subtypes α/β, α’/β’, and γ [64] but is enriched for α/β and γ relative to α’/β’. We collected female flies on the day of eclosion and dissected brains from *dSERT*^*4*^ *and dSERT*^*16*^. KCs from each genotype were dissociated in parallel and isolated via FACS using the GFP marker (Fig. 1B). 5 replicates per genotype were obtained and bulk RNA-seq libraries (SMART-seq) were prepared for all samples and sequenced together. PCA (data not shown) revealed two samples (one of each genotype) with increased technical variability; these were removed from subsequent analyses.

Differentially expressed genes (DEGs) between *dSERT*^*16*^ and *dSERT*^*4*^ samples were identified using DESeq2 [43], and revealed 44 upregulated and 54 downregulated (p_adj_ < 0.05) (Fig. 1C, D and Supp. Table T1). These include DEGs with functions that could represent homeostatic adjustments to perturbations in serotonergic signaling during development, such as transcription factors (*Lim1, Achi*), proteins involved in neuronal maturation and development (*Trim9, Mis12*) [65,66], a *Drosophila* ortholog of calbindin (*Cbp53E*), ion channels (*Ork1, Ppk29*), and other GPCRs (*Dh44-R1, Proc-R, CCHa2-R, Ir76a*) (Fig. 1C, D and Supp. Table T1). When genes were plotted by chromosomal position, however, there was a striking concentration of DEGs on the same arm of the 2^nd^ chromosome (chr2R) as the *dSERT*^*16*^ DNA lesion (Fig 1E). *Drosophila* have only 3 chromosomes that house most of their genome, and some of these observations may represent true findings. However, the buildup on chr2R suggests that at least some of the observations may derive from disruption of genomic DNA rather than changes in serotonergic signaling.

Though SMART-seq libraries feature increased sensitivity to lowly-expressed transcripts, they necessitate pooling of RNA from all cell-types within the collected population and may result in washout of cell-type specific changes. To investigate the transcriptomics of each KC subtype independently, we followed a recent single cell RNA-seq strategy in which all samples and replicates are pooled and processed together [38,44]. We generated *dSERT*^*16*^ and *dSERT*^*4*^ fly lines with GFP expressed in KCs as above, but included an additional element unique to each biological replicate: a 3^rd^ chromosome derived from independent WT strains available from the *Drosophila* Genetics Research Panel (DGRP) [45]. Because transcripts derived from DGRP chromosomes bear SNPs, single cells can be bio-informatically traced to genotype-of-origin *post-hoc* (Fig. 2A). This allowed us to pool all replicates of both control and mutant samples for dissociation, FACS, library prep, and sequencing, thereby minimizing long-standing issues of technical variability between individual replicates that contribute to bias in RNA-seq data. Dimensionality reduction (Supp. Fig. S1) resulted in robust clusters for two sub-populations for KC_α/β_ (KC_AB1, KC_AB2), two for KC_γ_ (KC_G1, KC_G2), and one for KC_α’/β’_ (KC_ABp1) (Fig. 2B). Running pseudobulk differential expression between mutant and control cells collapsed by cell-type revealed 33 significant changes. Some changes were cell-type specific (e.g. *SK* in KC_G1 and *CG31690* in KC_AB1), and many were observed in multiple cell-types (e.g. *prom, Cbp53E, CG42392, Pgant9*) (Fig. 2C, D and Supp. Table T2). For those DEGs that were identified as cell-type specific such as *SK*, we detected robust transcript expression in most of the clusters, lending credence to the hypothesis that the DE observed is in fact specific to a particular cell-type (Supp. Fig. S2). When visualized in pseudo-Manhattan plots (Fig. 2E), however, the bias of DEGs to chr2R was even more pronounced than for SMART-seq (Fig. 1E), highlighting their possible artefactual provenance. The DEGs on chr2R appear to lie in two positional “columns” – one ~7.5 Mb away from *dSERT*, and one that is immediately adjacent to the *dSERT*^*16*^ deletion. One of the DEGs immediately adjacent to the deletion is an eye-specific gene, *prom*, that is not expressed in WT KCs. By extension, we concluded that upregulation of the *prom* transcript in *dSERT*^16^ is likely to represent an artefact caused by the deletion of regulatory DNA adjacent to *dSERT* and *prom*.

To explore the possibility that more precise mutations in *dSERT* might be less disruptive and generate fewer artefactual hits, we generated a new mutant allele using CRISPR [67] to precisely excise ~2.6kb DNA coding for most of the first and second transmembrane domains and simultaneously induce a frameshift in the CDS. We reasoned that even if the resultant mRNA could code for a partial dSERT protein, it would be topologically inverted in the plasma membrane (Fig. 3A). Fly lines bearing the deletion, termed *dSERT*^*TMKO*^, were outcrossed six times to *w*^*1118*^. The presence of the deletion was confirmed by PCR-sanger sequencing, and behaviorally in that this line phenocopies the sleep deficit found in *dSERT*^*16*^ (data not shown). We then built fly lines as in the previous experiment, using the new *dSERT*^*TMKO*^ allele and second chromosomes derived from *w*^*1118*^ as controls, in place of *dSERT*^*16*^ and *dSERT*^*4*^, respectively. Sample prep, scRNA-seq, and data processing (Fig. 3B) were performed using the same pipeline as for the previous experiment. Again, relatively few (13) DE observations were made between mutant and WT cells (Fig. 3C, Supp. Table T3). However, in this dataset there is no pronounced enrichment of DEGs on chr2R (Fig. 3F). Importantly, some of the DEGs on chr2R in the previous (*dSERT*^*16*^) dataset, including those immediately adjacent to *dSERT*, such as *prom*, are absent from this *dSERT*^*TMKO*^ dataset (Supp. Fig. S2B). Some genes DE in this experiment were not detected in the previous dataset, such as *LysRS* in multiple cell types and *dpr1* and *mamo* in KC_ABp1 and KC_G2, respectively.

While it is known that KCs undergo extensive remodeling during pupation [68–72], most of the literature establishing the importance of serotonergic signaling onto them concerns behaviors such as sleep and memory, which are not utilized during pupation. We thus hypothesized that some of the transcriptional changes in response to *dSERT* LOF may not accumulate until the circuit undergoes perturbed activity in the adult fly brain. To assess transcriptional changes that may accumulate after eclosion, we repeated the *dSERT*^*TMKO*^ scRNA-seq in 4-6 day-old adult flies (Fig. 4A). This experiment yielded a lower cell number per cluster (Supp. Fig. S1F) than those using freshly-eclosed adults, limiting statistical power in calling DE. Nonetheless we observed a small number (15) DEGs between dSERT^TMKO^ mutant and WT cells (Fig. 4C,D and Supp. Table T4). Interestingly, some genes (e.g. *LysRS*) were shared with the previous (day 0) dataset, while *Cbp53E*, a gene identified in the *dSERT*^*16*^ day 0 dataset but not found in the *dSERT*^*TMKO*^ day 0, reappeared in this *dSERT*^*TMKO*^ day 4-6 dataset.

The use of constitutive *dSERT* deletion mutants ensures complete and specific SERT LOF, but it is not possible to distinguish between developmental and adult effects. As a first step to study the effects of long-term SERT blockade in circuits that develop normally, we fed adult flies 3mM citalopram (CIT) to pharmacologically inhibit SERT, a concentration that phenocopies the effect of the *dSERT*^*16*^ allele on sleep behavior [62]. After feeding WT flies either CIT or vehicle (VEH) from eclosion for 4-6 days (Fig. 5A), we again isolated GFP-tagged KCs and used single cell seq to assess DE. Similar to the previous two experiments, few genes (6 downregulated and 1 upregulated) were identified as DE across any KC subtype between CIT fed and control flies (Fig. 5B–D, Supp. Table T5). As predicted, there was no “pileup” of these observations on chr2R (Fig. 5E).

**Figure 6.**
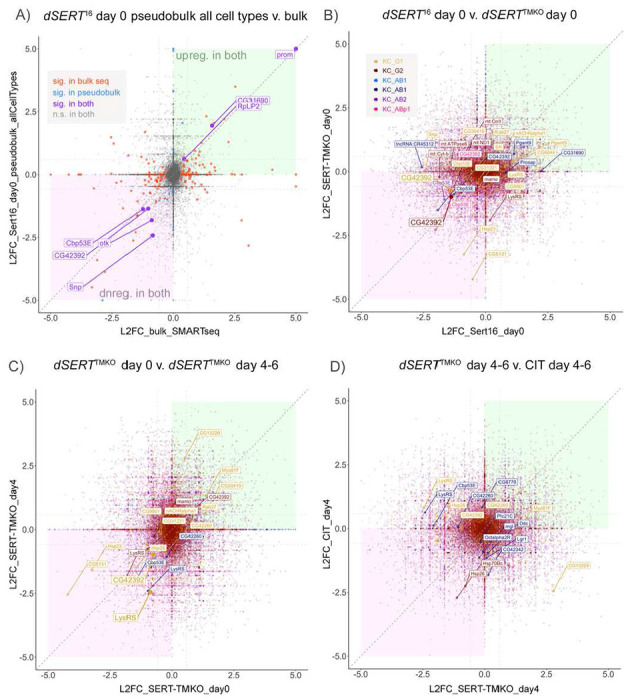
Correlation of genes identified as DE between datasets. A) Correlation plot showing log_2_ fold-change (L2FC) for DEGs in *dSERT^16^* versus *dSERT^4^* at day 0, comparing bulk sequencing (Fig. 1) and the initial scRNA-seq data (Fig. 2) analyzed using “pseudobulk” to collapse all clusters into one artificial “cell-type” for comparison with the bulk dataset. Concordant genes significant in both datasets are plotted in a larger font, and colored purple. Genes significant in only the bulk or scRNA-seq datasets are colored red or blue, respectively. Diagonal dark grey dashed line represents 1:1 correlation between datasets. The lighter grey horizontal and vertical lines represent 1.5 fold-change cutoffs for genes of interest. B) Correlation plot between *dSERT*^16^ and *dSERT^TMKO^* day 0 scRNA-seq datasets. Genes are color-coded by KC type as in previous figures. Genes not significant in either dataset are plotted with reduced opacity. Genes significant in at least one dataset are plotted with normal opacity. While there are many genes with L2FC of the same sign in both datasets, most are only significant in one dataset (smaller labeled points). C) Correlation plot comparing data derived from newly eclosed (day 0) vs aged flies (day 4-6) using the *dSERT^TMKO^*. Genes are plotted as in B. *CG42392* and LysRS in KC_G1 were significant in both datasets (larger labels and points), with DE in the same direction (down regulated). D) Correlation plot between aged *dSERT^TMKO^* (d4-6) and aged flies fed citalopram (CIT). One gene (*Hsp26*) was DE in both datasets, although in a different cell type in each dataset and therefore not highlighted.

To formally assess concordance between the five datasets, we constructed correlation plots displaying pairwise comparisons of the log_2_(fold-change) values for each DE observation. To compare our bulk RNA-seq for *dSERT*^*16*^ vs. *dSERT*^*4*^ with our first scRNA-seq experiment using the same alleles, we first collapsed all cell-types in the scRNA-seq into one and conducted “pseudobulk” analysis on the entire population of cells. Correlation between these two measures revealed that the bulk RNA-seq picked up many more DEGs (161) than “pseudobulk” from scRNA-seq (26) (Fig. 6A). Many genes, however, exhibited fold-change values of the same sign (up or downreg), even if p_adj_ was only significant in one dataset. Notably, several genes (*Cbp53E, otk, CG42392, Snp, RpLP2, CG31690*) were concordant between datasets, exclusive of those such as *prom* flagged as artefacts. Next, we compared the *dSERT*^*16*^ and *dSERT*^*TMKO*^ day 0 scRNA-seq datasets in a similar correlation plot, but retained the cell-type specific DE conducted in the original analysis (Fig. 6B). Again, most DE observations were significant in only one dataset (smaller labels), though *CG42392* was concordant and significant in KC_G1 and KC_G2 in both datasets. Comparison of the *dSERT*^*TMKO*^ day 0 and day 4-6 datasets similarly revealed only concordant changes that were significant in both datasets (Fig. 6C), *CG42392* and *LysRS* in KC_G1. Finally, comparison of the *dSERT*^*TMKO*^ day 4-6 and CIT-fed day 4-6 experiments showed no concordant changes that were significant in both datasets, but many that were significant in one (Fig. 6D).

## Discussion

We have tested whether specific subtypes of post-synaptic cells in a defined serotonergic circuit undergo transcriptional changes in response to the inhibition of dSERT. A large number of previous reports have investigated transcriptomic changes in response to SSRI-like perturbations, but most have used peripheral samples or highly heterogenous brain tissue as input. More recently, specific subtypes of neurons have been targeted using molecular-genetic strategies employed in rodents such as RiboTag [21,73] and untargeted scRNA-seq [14]. We have now employed similar strategies in the fly with an additional purification step – FACS sorting of GFP labeled cells to isolate a genetically-labeled neuronal subtype: the KCs of the mushroom bodies. We have also compared our DE results obtained across two independently-derived, *dSERT* mutant alleles, two different age groups, and against pharmacological SERT inhibition. Our efforts here focusing on KCs have uncovered a small number of possible DEG candidates and defined several experimental pitfalls to consider in the further analysis of serotonergic signaling in the fly. Since the molecular machinery for serotonergic signaling is conserved from flies to humans we speculate that future experiments using similar methods may complement experiments in rodents to determine how different serotonergic circuits respond to inhibition of SERT.

### Bulk RNA-seq

We initially used a high-sensitivity bulk RNA-seq method (SMART-seq) to profile changes in *dSERT*^*16*^ mutant vs. *dSERT*^*4*^ control animals and flies collected on the day they eclosed as adults from pupae (day 0). Since we used a bulk sequencing method, reads from different KC subtypes were analyzed as a group. PCA revealed strong separation of samples by genotype and the elimination of one set of slight outlier samples (data not shown). Standard data processing and calculation of DE revealed 98 DEGs (p_adj_ ≤ 0.05). We note that this number is too low for gene ontology (GO) or similar analyses available for *Drosophila* [74,75] (data not shown) and that gene set enrichment analysis (GSEA) is not readily available for *Drosophila* [76]. Importantly, the number of genes we identified is comparable to the number of changes in ribosome-loaded transcripts observed in specific mouse cell types after SSRI treatment, including serotonergic neurons of the raphe nucleus [73], S100a10 corticostriatal neurons [21] and the lower range (48-1243 DEGs) of an additional 27 brain regions recently analyzed in mice [14]. However, we also observed an enrichment of DEGs on chr2R, proximal to the *dSERT* locus, suggesting that their differential expression might be artefactual, and derived from the dysregulation of adjacent or distal DNA affected by the deletion, or perhaps genetic linkage.

### scRNA-seq

Studies using bulk RNA-seq methods such as SMART-seq are limited by the heterogeneity of the cell-types used for input. In addition, it is known that small differences in sample treatment, even in those processed simultaneously and in parallel, contribute significantly to noise in sequencing data. To address these concerns, we used a newly developed scRNA-seq protocol to “tag” different biological replicates with different DGRP chromosomes, thus allowing them to be processed as a single sample [38]. In the first of these experiments, we again used *dSERT*^*16*^ mutant and *dSERT*^*4*^ control flies at day 0 post-eclosion. We observed an even more pronounced enrichment of DEGs on chr2R proximal to the *dSERT* locus, further suggesting that relatively small changes in genetic architecture can significantly affect the detection of transcriptomic differences.

To avoid the chromosomal effects of the *dSERT*^*16*^ imprecise excision allele, we generated a new mutant allele using CRISPR/Cas9 (*dSERT*^*TMKO*^). In contrast to *dSERT*^*16*^, the *dSERT*^*TMKO*^ deletion does not include DNA upstream of the start codon that may be more likely to contribute to the regulation of transcription of adjacent genes. We repeated the scRNA-seq experiment at day 0 using *dSERT*^*TMKO*^ and found that most of the DEGs on ch2R suspected to be artifactual in the *dSERT*^*16*^ dataset were absent in the *dSERT*^*TMKO*^ dataset, including *prom*, an eye-specific gene 4.3kb upstream of *dSERT*. Together, the data shown in Figs. 2 and 3 indicate that mutations in *dSERT* and other genes used in further analyses should be carefully selected to minimize the disruption of chromosomal architecture.

Interestingly, one of the few DEGs identified in the *dSERT*^*TMKO*^ day 0 dataset was *dpr1* in KC_ABp1, a cell-adhesion molecule that may represent an adjustment to dysregulated circuit activity in the presence of aberrant serotonergic signaling. SERT is present in developing serotonergic neurons [77], and SSRIs can cause dysregulation of circuit wiring in mammals [78–80]. Additionally, *Drosophila* serotonergic neurons are remodeled and form new synapses in development [81]. Many cells that express 5-HTRs undergo significant changes in gene expression during this time [38,82] and are further refined by activity [83–85]. It is plausible that other factors involved in circuit formation and stabilization may be targets of homeostatic adjustments in response to altered extracellular serotonin.

### Adult versus developmental effects of SERT LOF

We hypothesized that loss of dSERT activity during both development and adulthood, rather than development alone, might further alter the DE profile. To test this, we repeated the scRNA-seq protocol using flies that had been aged for 4-6 days rather than freshly-eclosed (day 0). We again observed some changes across multiple cell types (i.e. *LysRS, CG42260*), as well as some that were cell-type specific. Among these, the cell surface recognition molecules *beat-IIa* and *side* DE in KC_G2 could, similarly to *dpr1* in KC_AB1 in the experiment with day 0 flies, represent homeostatic changes to maintain proper connectivity. However, the total number of DEGs seen in the aged flies was similar to that seen with newly eclosed flies.

To further explore the effects of dSERT inhibition in the adult, we fed WT flies the SSRI citalopram (CIT) or vehicle (VEH) for 4-6 days and repeated our scRNA-seq workflow. We uncovered a new set of DEGs, most of which were observed only in the major KC_α/β_ subtype (KC_AB1) and which did not show significant overlap with those detected using mutants. It is possible that off-target effects of CIT dominate these observations, and drug specificity may be tested in future experiments by feeding CIT to *dSERT* mutants. It is also possible that the decrease in SERT activity caused by citalopram was less pronounced than the complete block in activity caused by *dSERT*^*TMKO*^, thus reducing the change in serotonergic signaling and the subsequent effects on post-synaptic cells. Alternatively, the very low number of DEGs we detect in adult flies fed citalopram, as well as the relatively small difference in the number of DEGs in day 0 versus day 4-6 *dSERT*^*TMKO*^ may be consistent with the idea that serotonergic signaling during development exerts more significant changes than inhibition of SERT in the adult. Further genetic methods to knock out *dSERT* during development versus adult flies will be used to address this issue. We note that in mouse models, many effects on behavior seen with both SSRIs and mutants that perturb serotonergic signaling are primarily based on exposure during development [79,86–91].

### Cell-subtype-specific effects

Some of the DE observed our scRNA-seq experiments appeared to be specific to particular KC types. it is possible that these differences arise from the different expression profiles of 5-HTRs, including the enrichment of 5-HT1A on KC_α/β_ and 5-HT1B on KC_γ_. It is also possible that differences in the extent or source of serotonergic innervation of different KC subtypes contributed to these differences. Our data show that although the number of detectable changes in response to dSERT LOF is low in this system, even highly similar cell-types (KC subtypes) exhibit different changes in response to the same chronic perturbation. Recent results in mice suggest a similarly heterogenous response in subtypes of hippocampal neurons [14]. We suggest that further experiments in the fly will complement studies in mammals to determine the molecular mechanisms by which serotonergic drugs exert their effects on different subsets of neurons.

### Technical and experimental limitations

Across all of our single cell RNA-seq experiments, both during development and in the adult, the total number of DEGs was lower than those identified in the initial bulk RNA-seq experiment. In contrast to the single cell protocol, SMART-seq captures cells in a chaotropic agent that halts transcriptional dysregulation induced by cell injury and protects RNA from degradation. This difference, and/or differences in library prep methodologies between SMART-seq and *10x* 3’GEX may have led to better detection of DEGs in our bulk RNA-seq experiment. More generally, it is known that the advantages of scRNA-seq come at the cost of low sequencing depth per cell.

Several additional factors may contribute to the low number of DE genes we observed in single cell experiments, including relatively low numbers of cells in some clusters (Supp. Fig. S1F). Our power to detect DE was strongest in the clusters with the highest cell number (KC_AB1 and KC_G1) and more cells may be needed to detect subtle changes in gene expression in other subtypes. The stringent nature of our analyses may also have excluded some subtle or variable changes. The percentage of p-values that survived *Benjamini-Hochsberg* multiple comparison correction in each of our scRNA-seq “pseudobulk” analyses was between 2 and 8%. This represents a standard tradeoff in sequencing studies between the unbiased measurement of all genes in the genome at the statistical cost of multiple comparisons. Unfortunately, this also presents a significant barrier in all current studies attempting to identify less consistent or smaller changes. Finally, it is possible that sample prep methodology should be further refined for this type of investigation. For example, in future experiments we will consider alternative methods such as flash freezing tissue [92,93], which may result in a faster and cleaner sample prep with fewer artefactual changes.

In addition to a relatively small number of DEGs per experiment, comparing our datasets in correlation plots reveals relatively little overlap. This may suggest that genomic background and experimental variability have stronger effects on DE analysis between groups than the effects of dSERT LOF. The least favorable interpretation of this lack of overlap is that most of the DEGs we detected were “noise”, however the stringent statistical analysis suggests otherwise. Based on both the relatively small number of DEGs as well as the relatively limited overlap we observe across experiments, we speculate that the specific post-synaptic cells we chose to study (KCs) may not mount a large transcriptional response to changes in serotonergic signaling. Using the myriad of available drivers to label and isolate different cell types in the fly may reveal different cell types that show more robust transcriptional responses to mutation of *dSERT* or feeding SSRIs than we identified in KCs. In addition, while neuronal excitation and even the signaling cascades modulated by serotonin are known to be intimately linked to transcription [94–96], these pathways are also regulated by many other factors. Serotonergic signaling may only cause weak or microdomain-restricted changes in some pathways, and it is possible that the primary adaptive response to an increase in extracellular serotonin is post-transcriptional. Additional -omic strategies, notably ChIP-seq and ATAC-seq [97,98], have been used with great success from similar starting samples, and provide a complementary approach to RNA-seq in future studies.

### Candidate genes for further investigation

Despite the low number of observations in this study, those identified may represent a true response to the inhibition of dSERT and changes in extracellular serotonin. If so, they are novel. These include *Cbp53E*, an ortholog of *calbindin* known to affect axon branching in *Drosophila* [99], and *pgant9*, an enzyme involved in sugar-modification of proteins [100,101]. While further validation will be needed, we suggest that concordance across some datasets may justify further investigation of these and other DEGs. In *Drosophila*, testing the functional effects of perturbing candidate genes, rather than additional molecular methods such as RT-PCR or in situ hybridization, may be the most efficient path to testing their validity. The large number of mutants available in the fly as well as the low cost of generating new mutants underscore the power of this approach and its complementary use with RNA-seq studies compared to those conducted other model systems such as rodents.

## Data Availability

All raw data and Seurat objects generated in this study will deposited on GEO and will be made available upon publication. (Accession number to follow.) No new algorithms were developed in this work
